# Neoantigen Vaccine Delivery for Personalized Anticancer Immunotherapy

**DOI:** 10.3389/fimmu.2018.01499

**Published:** 2018-07-02

**Authors:** Yugang Guo, Kewen Lei, Li Tang

**Affiliations:** ^1^Institute of Bioengineering, École polytechnique fédérale de Lausanne (EPFL), Lausanne, Switzerland; ^2^Institute of Materials Science and Engineering, École polytechnique fédérale de Lausanne (EPFL), Lausanne, Switzerland

**Keywords:** neoantigen, cancer vaccine, cancer immunotherapy, vaccine delivery, *in vitro* transcribed mRNA, synthetic long peptide, dendritic cell, nanoparticle

## Abstract

Cancer neoantigens derived from random somatic mutations in tumor tissue represent an attractive type of targets for the cancer immunotherapies including cancer vaccine. Vaccination against the tumor-specific neoantigens minimizes the potential induction of central and peripheral tolerance as well as the risk of autoimmunity. Neoantigen-based cancer vaccines have recently showed marked therapeutic potential in both preclinical and early-phase clinical studies. However, significant challenges remain in the effective and faithful identification of immunogenic neoepitopes and the efficient and safe delivery of the subunit vaccine components for eliciting potent and robust anticancer T cell responses. In this mini review, we provide a brief overview of the recent advances in the development of neoantigen-based cancer vaccines focusing on various vaccine delivery strategies for targeting and modulating antigen-presenting cells. We discuss current delivery approaches, including direct injection, *ex vivo*-pulsed dendritic cell vaccination, and biomaterial-assisted vaccination for enhancing the efficiency of neoantigen vaccines and present a perspective on future directions.

## Introduction

Vaccines activating the immune system for prevention and treatment of infections and other diseases have made major impact in human healthcare. Cancer vaccines have been actively pursued and studied for decades with several successful examples that are now in the market (1). However, prophylactic cancer vaccines so far have been effective only for virus-related cancers, such as human papillomavirus-induced cervical cancers (2). Provenge (Sipuleucel-T), the only U.S. Food and Drug Administration-approved therapeutic cancer vaccine to date, has only had modest clinical effect for the treatment of prostate cancer (2, 3). Compared to other immunotherapies, such as checkpoint blockade and adoptive T cell therapy (ACT), most cancer vaccines fail to demonstrate notable clinical efficacy. One of the key obstacles to the development of an effective cancer vaccine is the difficulty in antigen selection (4). Traditionally, cancer vaccines are designed to target tumor-associated antigens (TAAs) as they are overexpressed in cancers and could be universal targets among patients of the same malignancy (4). However, TAAs are also present in normal tissues and vaccines against TAAs can potentially initiate central and peripheral tolerance responses leading to low vaccination efficiency or autoimmunity against normal tissues (1, 5).

Tumor-specific antigens, also termed as neoantigens, are derived from random somatic mutations in tumor cells and not present in normal cells (6, 7). Compared to those non-mutated self-antigens, neoantigens could be recognized as non-self by the host immune system and are thus attractive targets for immunotherapies with potentially increased specificity, efficacy, and safety (4). The immunogenicity of neoantigens leading to T cell response has long been demonstrated in human (8). In fact, a number of preclinical and clinical studies have shown that neoantigen-specific cytotoxic T lymphocytes (CTLs) represent the most potent tumor-rejection T cell populations (9–12). However, naturally occurring neoantigen-specific CTLs in patients are typically rare likely because of low clonal frequency and inefficient presentation of neoantigens (13, 14). Therefore, cancer vaccine or ACT is necessary to potentiate potent immunity against neoantigens for cancer immunotherapy.

Recently, three independent clinical studies provided solid evidence that neoantigen-based cancer vaccines could be developed to elicit potent neoantigen-specific T cell responses against late stage melanoma with remarkable safety and efficacy (15–17). These and other recent advances (listed in Table 1) have triggered the enthusiasm in pursuing cancer vaccines against neoantigens. Many efforts are currently focused on addressing two key challenges in the development of neoantigen-based cancer vaccines for wide clinical applications. First, immunogenic neoantigens are rare and difficult to predict. Current predictive algorithm and validation tools need to be optimized for accurate prediction of major histocompatibility complex (MHC)-binding peptides and reliable selection of highly immunogenic neoepitopes (18). Second, it remains challenging to develop an universal and effective delivery strategy to target neoantigen-based vaccines to professional antigen-presenting cells (APCs) for eliciting robust and potent T cell responses against cancer (14). In this mini review, we summarize and discuss the recent progress in addressing these issues for the development of neoantigen-based cancer vaccines with an emphasis on various delivery strategies.

**Table 1:** Recent examples of neoantigen vaccine delivery.

Status	Indication	Antigen	Adjuvant	Route	T cell responses		
						
					CD4^+^	CD8^+^	Reference
**1. Direct injection of unformulated neoantigen vaccines**							
Phase I	Melanoma (stage III and IV)	mRNA	None	i.n.	0.1–2.0%^a^	0.02–0.55%^a^ 0.03–1.9%^b^	(16)

Phase I	Melanoma (stage IIIB/C and IVM1a/b)	SLP	Poly-ICLC	s.c.	0.03–0.06%^a^ 0.001–0.05%^b^	0.2–1.2%^c^	(17)

Preclinical study	MC-38 colon cancer	SLP	CD40 antibody and poly (I:C)	i.p.	NM	0.18–1.4%^a^ 0.48–1.33%^b^	(19)

Preclinical study	B16F10 melanoma	SLP	Poly(I:C)	s.c.	1.54%^c^	3.61%^c^	(20)

Preclinical study	d42m1-T3 sarcoma	SLP	Poly(I:C)	s.c.	NM	2.8–17.5%^b^	(21)

Preclinical study	A2.DR1 sarcoma	SLP	CFA, montanide-ISA51, and imiquimod	s.c.	1.91%^b^	NM	(22)

Preclinical study	B16F10 melanoma	SLP	Poly(I:C)	s.c.	NM	NM	(23)

***2. Ex vivo*-pulsed dendritic cell (DC) vaccine**							

Phase I	Melanoma (stage III)	*Ex vivo* SLP pulsed DCs	Poly(I:C), R848	i.v.	NM	0.06–0.9%^a^	(15)

**3. Biomaterials-assisted neoantigen vaccines**							

Preclinical study	B16F10 melanoma, 4T1 breast cancer, and CT26 colon cancer	mRNA-lipoplex	None	i.v.	1.36%^c^	1.67%^c^	(20)

Preclinical and phase I study	CT26 colon cancer, TC-1, and melanoma	mRNA-lipoplex	None	i.v.	NM	30–60%^a^, 0.62%^a^	(24)

Preclinical study	MC-38 colon cancer and E6/7-TC-1 lung cancer	SLP/PC7A nanoparticles	None	s.c.	NM	NM	(25)

Preclinical study	MC-38 colon cancer and B16F10 melanoma	SLP/nanodiscs	CpG	s.c.	~14.0%^c^	~30%^a^	(26)

Preclinical study	B16F10 melanoma	Endogenous neoantigen-containing proteins	None	s.c.	1.0–3.0%^c^	1.5–12%^c^	(27)

Preclinical study	E7-TC-1 lung cancer, B16F10 melanoma, and CT26 colon cancer	SLP/mesoporous silica microrod with PEI	CpG, PEI	s.c.	~0.6%^c^	~2.0%^a^ 1.5%^c^	(28)

Preclinical study	MC-38 colon cancer	SLP/DNA-RNA nanocapsule	CpG	s.c.	NM	9.5%^a^	(29)

*^a^Percentage of neoantigen-specific CD4^+^ (or CD8^+^) T cells among total CD4^+^ (or CD8^+^) T cells in peripheral blood or spleen detected by multimer staining or the Enzyme-Linked ImmunoSpot (ELISPOT) assay*.

*^b^Percentage of neoantigen-specific CD4^+^ (or CD8^+^) T cells among total CD4^+^ (or CD8^+^) T cells in tumor detected by multimer staining*.

*^c^Percentage of neoantigen-specific CD4^+^ (or CD8^+^) T cells among total CD4^+^ (or CD8^+^) T cells in peripheral blood or spleen detected by intracellular interferon-γ (IFN-γ) staining*.

*i.n., intranodal injection; s.c., subcutaneous injection; i.v., intravenous injection; i.p., intraperitoneal injection; NM, not measured; poly-ICLC, polyriboinosinic-polyribocytidylic acid-polylysine carboxymethylcellulose; poly(I:C), polyinosinic:polycytidylic acid; SLP, synthetic long peptide; CFA, complete Freund’s adjuvant; CpG, unmethylated cytosine-phosphate-guanine oligodeoxynucleotides; PEI, polyethyleneimine*.

## Identification and Selection of Neoantigens

**Figure 1:**
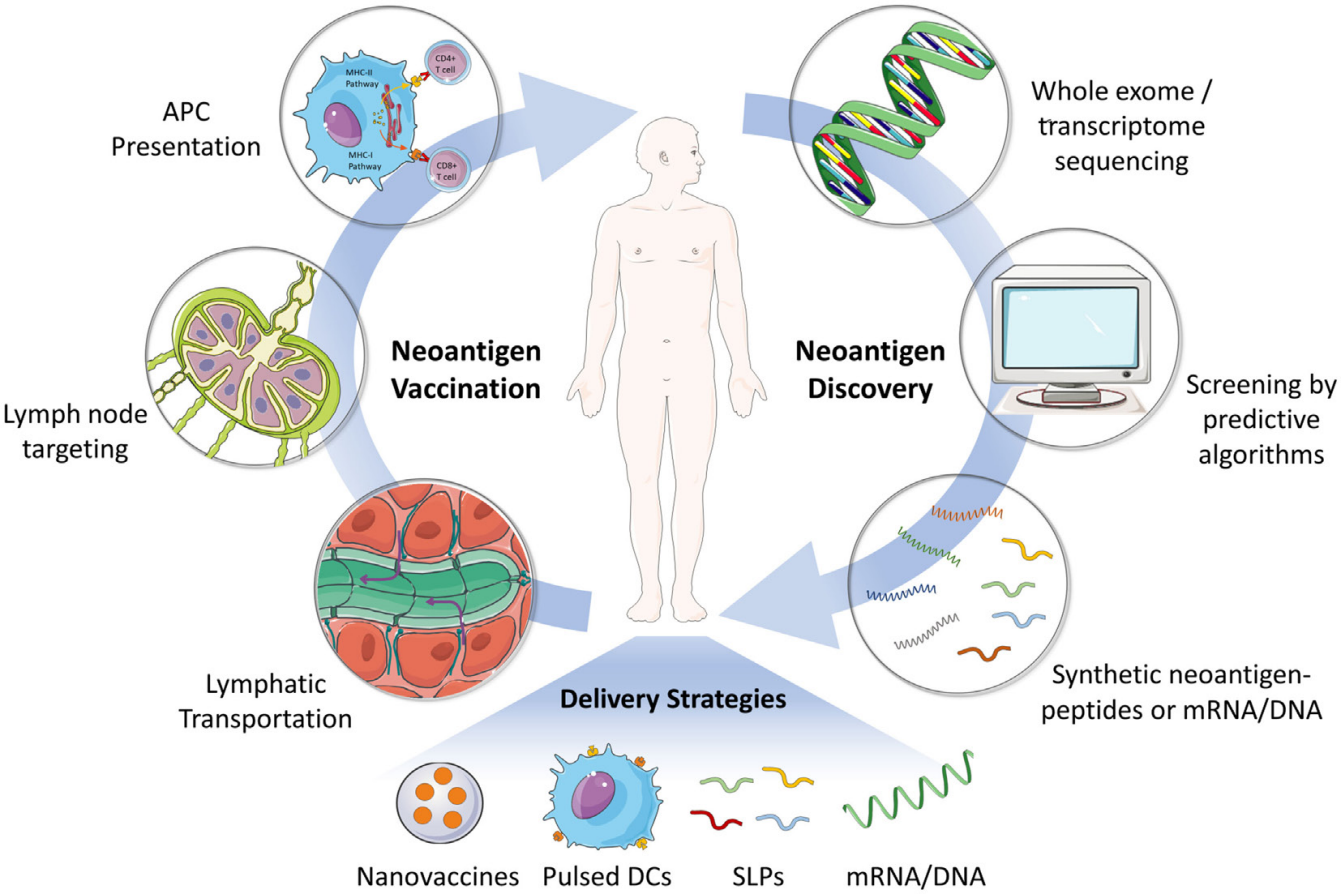
Schematic illustration of the process of neoantigen discovery, vaccine manufacturing and formulation, and vaccination in patients. The first step for developing neoantigen cancer vaccine involves the identification of mutated tumor specific antigens by whole exome/transcriptome sequencing and prediction of immunogenic MHC epitopes. Next, neoantigen vaccines (e.g., SLP and mRNA) are manufactured and formulated for efficient delivery to secondary lymphoid organs (e.g., lymph node), where neoantigen vaccines are captured by APCs and presented to effector immune cells including CD8^+^ or CD4^+^ T cells. Various delivery strategies have been developed to achieve an effective and safe neoantigen-based cancer vaccine. Abbreviations: SLP, synthetic long peptide; DC, dendritic cell; APC, antigen-presenting cell; MHC, major histocompatibility complex.

Neoantigen-related immunotherapy is a truly personalized therapy because most neoantigens are derived from unique mutations in each tumor genome (2). Therefore, identification of patient-specific immunogenic neoantigens is the first step in developing such personalized vaccines (Figure 1) (5). With the recent advances in genome sequencing technology as well as the MHC epitope database and predictive algorithms, it now becomes possible to identify and screen cancer neoantigens for individual patients (4, 5). In general, tumor or tumor-related samples are subjected to whole exome or transcriptome sequencing (2, 30). Non-synonymous somatic mutations in cancers, such as point mutations and insertion–deletions, could be identified by comparing the sequences of tumor and matched healthy tissues. Next, the discovered mutations are screened using predictive algorithms for MHC peptide binding affinity in order to identify the most immunogenic antigen candidates for manufacturing personalized cancer vaccines (5, 31). Currently, there are many predictive algorithms available for the identification of potential high-affinity binders of MHC class I molecule. However, the reliability of these predictive algorithms still needs to be improved (32). Most of the existing programs are not able to take into account every factor that impacts immunogenicity, for example, peptide processing by the proteasome, MHC binding stability, genetic insertion–deletions, or fusions, and so on (5). In addition, there are far less data available for predicting MHC class II restricted antigenic peptides (4, 30).

Other methods are also exploited currently to identify the cancer neoantigens besides sequencing of tumor samples. For example, mass spectrometry analyses of peptides from the peptide–human leukocyte antigen (HLA) complex have enabled the discovery of HLA ligandome tumor antigens for personalized vaccines (33–35). New strategies based on the functional analyses of peripheral blood mononuclear cells or tumor filtrating lymphocytes are being developed to identify neoantigen-reactive T cells (12, 36). These assays aiming to identify the pre-existing neoantigen-reactive T cells may fail to detect the subdominant and/or dormant neoantigens that do not elicit naturally occurring immune responses but are potentially important therapeutic targets.

## Delivery Strategies for Neoantigen Vaccines

A large number of approaches have been developed for the preparation, formulation, and delivery of different cancer vaccines, for example, whole tumor cell lysate-, nucleotide (mRNA/DNA)-, protein or peptides-based vaccines, dendritic cell (DC)-based vaccines, viral vectors, biomaterial-assisted vaccines, and so on (1, 2). In the context of neoantigen-based cancer vaccines, mRNA/DNA or synthetic long peptides (SLPs) are typically employed (Figure 1) (32). However, it remains challenging to develop a general method for the efficient delivery of these subunit vaccines for stimulating potent antitumor T cell responses (1, 14).

In general, parenterally injected soluble subunit antigens or molecular adjuvants rapidly disseminate into systemic circulation due to their small molecular sizes and show very poor targeting and accumulation in draining lymph nodes (LNs) resulting in limited immune response (37–39). Moreover, soluble molecular adjuvants administered subcutaneously often cause significant systemic inflammatory toxicities (39–41). To solve this problem, vaccines were administered in "depot"-based adjuvants, such as incomplete Freund’s adjuvant. However, these passive depots of antigens likely lead to tolerogenicity rather than immunogenicity (42). In addition, the therapeutic cancer vaccine is expected to elicit robust CD8^+^ T cell responses, which is essential to act synergistically with CD4^+^ T cell responses to destroy tumors (43). This presents another significant challenge for neoantigen vaccine delivery as typically only live infections induce potent CD8^+^ T cell priming (44). Soluble tumor antigens acquired by DCs are trapped in endolysosomal compartments and digested into peptides, which are subsequently loaded almost entirely onto MHC class II molecules for presentation to CD4^+^ helper T cells. In contrast, only the antigen peptides in cytosol are processed and loaded onto MHC class I molecules for the presentation to CD8^+^ killer T cells (44). Thus, it is also critical in neoantigen vaccine design to achieve cytosol delivery of those antigens for effective cross-priming of CTL responses (45).

To date, several different delivery strategies have been developed for neoantigen vaccines in preclinical and clinical studies, including direct injection of unformulated antigens, DC-based delivery strategy, and biomaterial-based delivery systems (Table 1). Here, we give a brief overview of various strategies and discuss their pros and cons.

### Direct Injection of Unformulated mRNA Vaccines Encoding Neoepitopes

*In vitro* transcribed (IVT) mRNA has undergone many preclinical and clinical investigations for therapeutic cancer vaccination with the advantages of self-adjuvanting activity, direct translation into the cytoplasm, low risk of insertional mutagenesis, as well as simple and inexpensive manufacturing procedure (46). However, controlling the translational efficiency of IVT mRNAs remains challenging. Unformulated mRNA could be spontaneously taken up by many kinds of cells through scavenger receptor-mediated endocytosis. As a result, only a small part of administered mRNA could be captured by APCs and reach cytoplasm for subsequent translation and antigen presentation. In order to maximize the capture of antigens by APCs, unformulated IVT mRNA can be administered directly into LNs through ultrasound-guided percutaneous injection [noted as intranodal (i.n.) injection], a clinically applicable administration route for the direct access to inner organs or tissues through needle-puncture of the skin (47).

Most recently, Sahin and his group demonstrated an elegant example of immunizing advanced melanoma patients in a clinical study using vaccines based on synthetic mRNAs encoding poly-neoepitopes through i.n. injection (Table 1) (16). This administration route improved the stability and translation efficiency of the IVT mRNAs, and enhanced the presentation of the neoantigens with MHC class I and II molecules on DCs. These IVT mRNAs also promoted DCs maturation *via* TLR7 signaling pathway due to intrinsic adjuvant capability. Potent T cell responses against multiple neoantigens were successfully induced in all the patients after immunization. It is noticeable that the majority of neoantigen-elicited T cell responses were HLA class II restricted CD4^+^ T cell responses even though they were predicted as high-affinity HLA class I binders. Although promising, the i.n. administration method may limit its wide application in many vaccination settings as the extensively repeated percutaneous injection (up to 20 vaccinations used in this study) may not always be practical.

### Direct Injection of Unformulated SLP Neoantigens

Antigenic peptide has been extensively exploited for cancer vaccines as it presents several advantages including direct function as pivotal T cell epitope, low toxicity, low cost, and ease of synthesis (48, 49). In a pioneered phase I clinical study evaluating SLP-based neoantigen cancer vaccines, a selected pool of twenty SLPs (15–30 mers for each peptide) together with adjuvant (poly ICLC) were used to immunize 6 patients with advanced cutaneous melanoma (Table 1) (17). During the treatment, seven vaccine doses were administrated through subcutaneous (s.c.) injection within 20 weeks. These peptide-based neoantigen vaccines induced polyfunctional MHC class II restricted CD4^+^ T responses targeting ~60% of neoantigens used across patients, while the induced MHC class I restricted CD8^+^ T cells targeted ~16% of those neoantigens. Encouragingly, four of six vaccinated patients were cancer-free 25 months post treatment. Similarly, in a recent preclinical study, mice immunized intraperitoneally (i.p.) with three neoantigen SLPs together with adjuvant showed potent therapeutical CD8^+^ T cell responses against MC-38 tumor with complete inhibition of tumor growth in 11 of 15 vaccinated mice (19).

However, subcutaneously administered peptide-based vaccines could rapidly diffuse into the peripheral blood vessels leading to systemic dissemination due to the relatively small molecular sizes (14, 49, 50). The ultimate therapeutic efficacy of these peptide vaccines is limited by inefficient delivery to desired lymphoid organs. Increasing dose or dosing frequency could partly solve this problem but in turn increases the risk of systemic toxicity. Intradermal vaccination strategies for SLPs have been tested in some clinical trials to successfully stimulate antigen-specific T cell responses with a low dose of SLPs (51), and thus could potentially serve as an alternative.

### *Ex Vivo*-Pulsed DC Vaccine

Neoantigens could also be delivered by DCs, which play a key role in antigen presentation in the immune system. Similar as Sipuleucel-T, DC vaccines targeting neoantigens have been developed and evaluated in a small-scale clinical trial (15). Patients’ monocyte-derived immature DCs were first matured through co-culturing with irradiated feeder cells in the presence of adjuvants and then separately pulsed with different SLPs for loading on the HLA class I or II molecules. Three patients with advanced melanoma received the adoptive transfer of peptide-pulsed mature DCs *via* intravenous (i.v.) infusion (Table 1) (15). It was found that this vaccine increased the preexisting neoantigen-specific immune response and promoted a diverse patient-specific TCR repertoire against previously undetected HLA class I restricted neoantigens.

In addition to peptides, IVT mRNAs have been utilized to transfect DCs for the generation of DC-based vaccines in many preclinical and clinical studies (51, 52), and are potentially useful for preparing DC-based neoantigen vaccines (53). In general, although proven to be effective and safe in clinical trials, the approach of *ex vivo*-pulsed DC vaccine is costly, labor-intensive, and requires highly skilled technicians for manufacturing, which greatly limits its wide clinical applications in a large scale (54).

### Biomaterial-Assisted Neoantigen Vaccines

Biomaterials have been extensively investigated for vaccine delivery as they could protect antigen and adjuvant molecules from degradation, enhance lymphoid organ targeting, and modulate APCs’ functions. Biomaterial-assisted cancer vaccines have shown great potential in both preclinical and clinical development (55–57). For example, a scaffold-based vaccine is being evaluated in phase I clinical trial (NCT01753089) for preventing melanoma. Neoantigen-based cancer vaccine delivery with biomaterials is a nascent area (Table 1) (14). Rapid progress has been made in designing novel biomaterials to deliver mRNA- or SLP-based neoantigens in tandem with adjuvants for enhanced cancer vaccines (20, 24).

Biomaterial delivery systems have been employed to improve the efficacy of peptide- or mRNA-based neoantigen vaccines. For example, a responsive nanovaccine was developed by self-assembling peptide neoantigens with ultra-pH-sensitive polymers (25). Such nanovaccines could achieve efficient cytosolic delivery of antigens in response to the acidic pH in endosomes leading to enhanced cross-presentation. Interestingly, this nanovaccine is adjuvant-free and the carrier polymer itself serves as an adjuvant *via* the stimulation of STING pathway (25). By tuning the chemical structure of the side chains of the polymers for the optimized transition pH, the nanovaccines could induce robust antigen-specific CTL responses with comparable or better efficacy than several established adjuvants [e.g., alum and unmethylated cytosine-phosphate-guanine (CpG) oligodeoxynucleotides]. Also, the CTL responses were type I interferon (IFN) pathway dependent as the majority of CTL responses were abolished in IFN receptor knockout (IFN-α/βR^−/−^) mice. As the micelle-based nanovaccine does not require any chemical modification of the peptide antigens, it could be easily adapted for different peptide antigens. In another elegant example, synthetic high-density lipoprotein nanodisc, a highly clinically safe and scalable material, was used to promote the co-delivery of peptide neoantigen through disulfide conjugation and cholesteryl-modified adjuvant to draining LN for prolonged antigen-presentation (26). The nanodisc elicited extremely high level (~30%) of antigen-specific CTL responses leading to eradication of established tumors when combined with checkpoint blockade antibody treatment.

Despite the technical challenges of systemic delivery of subunit vaccines (58), a recent study has been able to demonstrate a remarkably high delivery efficiency of IVT mRNA neoantigen vaccines into systemic DCs using lipid complex (Table 1) (24). Net charge of the RNA-lipoplexes (RNA-LPX) was found essential for the spleen targeting. When the charge ratio was optimized (+/− = 1.7/2–1.3/2), the model antigen was almost exclusively delivered and expressed in splenic cell populations. It is also noticeable that no molecular targeting ligands were used to modify the RNA-LPX surface. CD11c^+^ conventional DCs in the marginal zone, and plasmacytoid DCs and macrophages in the spleen were found to internalize the most RNA-LPX; those DCs were also found effectively translate the delivered mRNAs. Such RNA-LPXs encoding neoepitopes induced unusually high level of antigen-specific CTL responses (up to 30–60% among the total CD8^+^ T cell population). The potent effector and memory T cell responses together with IFN-α-mediated innate immune response effectively eradicated murine CT26 lung tumors (i.v. inoculated). The remarkably high efficiency of systemic APC targeting mediated by the negatively charged lipid complex is likely the reason for such potent elicitation of antigen-specific CTL responses.

In addition to actively targeting vaccines to LNs, biomaterials have also been designed for passive delivery *via* antigen capture *in vivo* (27). To prove this concept, poly(lactic-co-glycolic acid) nanoparticles with various surface modifications were developed to capture the tumor-derived antigens *in situ* post radiation therapy that induced immunogenic cell death. The capture efficiency could be fine-tuned by varying the surface chemistry of nanoparticles. Intratumorally injected nanoparticles captured released tumor antigens including neoantigens, and facilitated the internalization and presentation of tumor antigens by APCs. Such antigen-capturing nanoparticles substantially increased the ratio of tumor-infiltrating effector CD8^+^ T cells to regulatory CD4^+^ T cells. This *in situ* local vaccination strategy is facile and intrinsically personal. It also showed enhanced abscopal antitumor effect by inducing systemic immunity in mouse models.

Besides nanosized biomaterials, bulk biomaterials can also be utilized for enhancing cancer vaccines through constructing artificial antigen-presenting niche *in vivo* (59). Such artificial niche is designed to recruit DCs for antigen capture and presentation and activate DCs *in situ* (60). One very recent example is a scaffold-like neoantigen vaccine made from mesoporous silica microrods (MSRs) (28). A cationic polymer, polyethyleneimine (PEI), was coated onto MSRs for the adsorption of neoantigens on the scaffold. Interestingly, PEI itself could stimulate DCs with increased expression of CD86, and production of interleukin-1β (IL-1β) and tumor necrosis factor-α. CpG and granulocyte–macrophage colony-stimulating factor were loaded on the scaffold surface to serve as vaccine adjuvant and DC-recruiting factor, respectively. Impressively, when loaded with a pool of B16F10 or MT26 neoantigens, this scaffold vaccine eradicated the lung metastases and synergized with anti-CTLA4 antibody inducing regression of subcutaneous tumors in mice. This simple and modular strategy without chemical modification of the peptide neoantigens has great potential to enable robust personalized vaccination.

## Future Outlook

Vaccination against neoantigens has already demonstrated tremendous potential in both preclinical and clinical studies. As illustrated by diverse examples in this review, various vaccine delivery strategies, in particular, novel biomaterial-assisted vaccines, have shown great promise to elicit potent T cell responses for cancer treatment. Despite the rapid advances, enormous challenges remain for the future development of neoantigen-based cancer vaccines for wide clinical applications. So far, most clinical and preclinical studies using neoantigen vaccines have been focused on cancers with high mutation load; the feasibility of applying this approach to cancers with relatively low mutation rate is to be demonstrated (61). It also remains challenging to identify and select the immunogenic neoantigens from an individual’s tumor for enhanced therapeutic efficacy.

A general efficient and safe delivery strategy for neoantigen vaccines is still lacking. Innovative delivery strategies are continually being pursued by scientists to address this issue. *Ex vivo*-pulsed DC vaccines are promising but suffer from several limitations including the difficulty in preparation and expansion. Alternative cells are currently under development, such as B cells, which are promising APCs with much higher abundance than DCs, improved proliferation capability, and increased lymphoid organ targeting properties (62). Another promising strategy is using synthetic APCs that mimic the functions of natural APCs and are much easier to manufacture (63).

Rationally designed biomaterials are of particular interest to boost the development of neoantigen vaccines as they could be engineered exquisitely to fulfill all the delivery requirements. These biomaterials should be highly biocompatible, facile in preparation requiring minimum modification of the antigen itself, and highly modular for various neoantigens. Biomaterials based carriers are expected to achieve the co-delivery of several to tens of exogenous neoantigens together with adjuvants to target APCs, which are necessary for eliciting potent and broad T cell responses to prevent tumor escape in the clinic (16, 17, 20). Biomaterials are particularly useful to modulate intracellular delivery and antigen processing in APCs. Intelligent biomaterials are also expected to achieve precise control of balanced MHC class I and II loading of antigens for eliciting the most potent antitumor immunity.

## Author Contributions

YG, KL, and LT wrote and revised the manuscript.

## Conflict of Interest Statement

The authors declare that the research was conducted in the absence of any commercial or financial relationships that could be construed as a potential conflict of interest.
